# Exploring the Multifaceted Genus *Acinetobacter*: the Facts, the Concerns and the Oppoptunities the Dualistic Geuns *Acinetobacter*

**DOI:** 10.4014/jmb.2411.11043

**Published:** 2025-02-25

**Authors:** Tsvetana Muleshkova, Inga Bazukyan, Konstantinos Papadimitriou, Velitchka Gotcheva, Angel Angelov, Svetoslav G. Dimov

**Affiliations:** 1 Sofia University “St. Kliment Ohridski”, Faculty of Biology, Department of Genetics, 8, Dragan Tzankov blvd., 1164 Sofia, Bulgaria; 2 Yerevan State University, Faculty of Biology, Department of Biochemistry, Microbiology and Biotechnology, 1, Alex Manoogian str., 0025 Yerevan, Armenia; 3 Agricultural University of Athens, Department of Food Science and Human Nutrition, Laboratory of Food Quality Control and Hygiene, Iera Odos 75, Athina 118 55, Greece; 4 University of Food Technologies in Plovdiv, Faculty of Technology, Department of Biotechnology, 26, Maritza blvd., 4002 Plovdiv, Bulgaria; 5 Center of Competence "Agrofood Systems and Bioeconomy”, 26, Maritza blvd., 4002 Plovdiv, Bulgaria

**Keywords:** *Acinetobacter* genus, pathogenic and non-pathogenic species and isolates, genetic divergence

## Abstract

In recent years, the research community has been interested in members of the *Acinetobacter* genus mainly because of their role as causative agents of nosocomial infections. However, this rich-in-species genus has been proven to play a significant role in several biotechnological processes, such as bioremediation and fermented foods production. To partially fill the lack of information on *Acinetobacter*’s dualistic nature, in this review, based on literature data, we attempt to summarize the available information on the different roles the members of the genus play by considering their genetic constitution and metabolic properties. We analyzed reports of genetic divergence between the pathogenic and non-pathogenic species and isolates, which can be explained by their high adaptability to the different ecological niches. In turn, this adaptability could result from intrinsic genetic variability due to mechanisms of horizontal genetic transfer, as well as high mutability determined by the expression of error-prone DNA polymerases. Yet, we concluded that further studies are needed, especially whole-genome sequencing of non-pathogenic isolates, which for the moment are relatively scarce.

## Introduction

The genus *Acinetobacter* is a part of the family *Moraxellaceae*, order *Pseudomonadales*, and represents a group of Gram-negative, typically aerobic, non-motile, non-fastidious, catalase-positive, oxidase-negative bacteria [1]. They are widely distributed in various environments, including fertile and non-fertile soil (emphasizing that the nutrient content is not heavily relevant), fresh and sea waters, solid wastes, fruits and vegetables, and foods with animal origins – meat, fish, honey and dairy products [2-4]. Their presence on the human skin and gut microbiome is notable, as is their occurrence in other animals - cattle, pigs, honeybees, and more [5, 6]. These bacteria are distinguished for their metabolic versatility and adaptability to these diverse ecological niches. Such qualities of the genus can be benefited from, as various strains can produce several economically valuable secondary metabolites, including hydrolase enzymes, bioemulsifiers and a range of biopolymers [7]. That would suggest their possible biotechnological application, which relies on their ability to biodegrade oil, xenobiotics and halogens, remove phosphate and heavy metals in wastewaters, and potentially produce industrially important bioproducts [8].

In recent years, *Acinetobacter* species have garnered significant attention due to their peculiar role in both public health and food technology, and therefore serving as a bridge between the two primary concepts by being easily transferred between the general and clinical environments. Evidence suggests that the presence of those bacteria in raw food such as vegetables and fruits and dairy products might cause the consumer to have a foodborne disease, among others, typically diarrhea. Their ability to do so is not very well determined, as many associate the disorder’s origin with the usual foodborne pathogens, such as *Staphylococcus aureus* or *Escherichia coli* [9]. Certain species are implicated in severe healthcare-associated infections, characterized by their remarkable ability to acquire resistance to multiple antibiotics [10, 11].

**Fig. 1:**
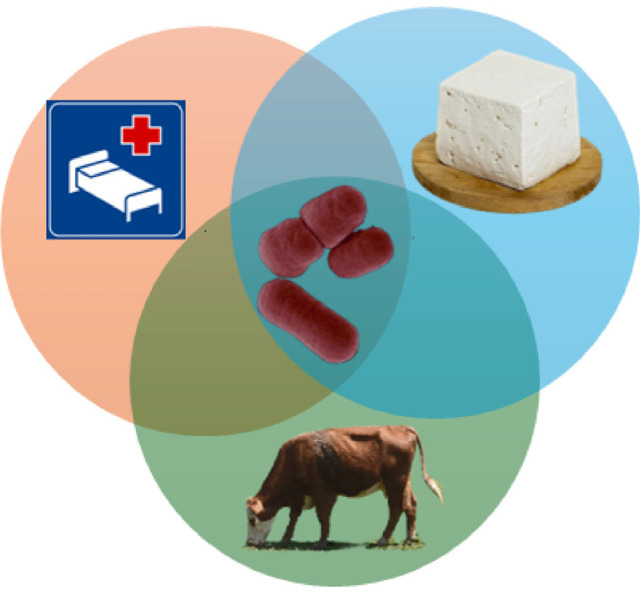
A diagram summarizing the relationships between *Acinetobacter*'s clinical impact, environmental roles, and biotechnological applications.

The common antimicrobial resistance genes present in the genus include class D oxacillinases like OXA-23, OXA-24/40, OXA-58, and the inherent OXA-51 in *Acinetobacter baumannii*, which are responsible for hydrolyzing β-lactam antibiotics, including carbapenems. Multiple drug resistance efflux pumps (AdeABC, AdeIJK, and AdeFGH), aminoglycoside-modifying enzymes (aph(3’)-VIa, AAC(6’)I-ad, and ArmA) and colistin resistance genes (mutations in *pmrA* and *pmrB*) are all present to a notable extent in various isolates, with the pathogenic ones being most relevant [12]. Due to the immense ecological spread of the genus *Acinetobacter* members, these bacteria have the potential to further escalate the transfer of antimicrobial resistance genes throughout the environment [13-15] (Fig. 1).

Contrarily, in parallel to the genus’s pathogenic nature, some non-pathogenic species could play beneficial roles in the fermentation of foods, contributing to flavor development and preservation. A study reported high *Acinetobacter*-related proteolytic activity, detected in 13 samples from the 15 taken from the Koozeh cheese, traditional for west Azerbaijan [3]. An examination of some types of French cheese made explicitly from raw goat’s milk concluded that *A. baumannii* accounted for 3.2% of the total isolated strains from the milk and rind during ripening [16]. *Acinetobacter johnsonii*, along with another unidentified representative of the genus, was noticed in French Livarot cheese both on its surface and core [17]. The genus was predominant in the ripening of Grana-like hard cheese made from raw cow’s milk, in the Piedmont region in Northwest Italy [18]. These observations could raise speculations that the presence of these bacteria in foods, especially in fermented ones, results from poor hygiene and lack of sanitary regulations in their production, therefore implying that detection of *Acinetobacter* is not recommended for the final product. While that might be an explanation, other findings suggest that the genus might possess certain characteristics that could positively impact the finished food product [3, 19]. Unfortunately, until now, studies focused on the functional roles of *Acinetobacter* species in food fermentations, as well as those exploring the transfer mechanisms within the food microbiotas, are rather scarce, and general trends cannot be stated.

## The Dualistic Nature of the Genus *Acinetobacter*

Genus *Acinetobacter* was first described in 1954 by Brisou and Prévot, who recognized the distinct characteristics that separated these organisms from other Gram-negative bacteria [20]. Initially, the genus included only a few strains, but subsequent advances in molecular biology techniques, particularly DNA-DNA hybridization and 16S rRNA gene sequencing, have further expanded the understanding and classification of this group [21]. As of today, the genus *Acinetobacter* contains over 80 recognized species [22]. These bacteria have a DNA G+C content of between 39 and 47%, according to Bergey’s Manual of Systematic Bacteriology [23, 24] and are divided into several complexes and subgroups based on their genetic, biochemical, and phenotypic characteristics. However, other authors recently reported that G+C content is between 37.2 and 45% [25], the disparity reflecting the broad genetic diversity between the different species. These complexes include the *Acinetobacter calcoaceticus* – *A. baumannii* complex, which is the most clinically significant group [26]. Other commonly explored species are *Acinetobacter lwoffii* and *A. johnsonii*, which are less associated with pathogenicity and more with their presence in the environment, various foods, and in the natural microbiota of humans and animals [21].

*Acinetobacter* species typically appear in pairs under a microscope and are known for their distinctive coccobacillary morphology, especially on non-selective agar media [1]. The cells of these bacteria differ in size and arrangement, usually from 0.9 to 1.6 μm in diameter and from 1.5 to 2.5 μm in length in the exponential growth phase.

They can use different carbon sources for their growth. When grown on non-differential solid media, *Acinetobacter* species usually form smooth or mucoid colonies ranging from white to pale yellow or light grey [27].

One of the genus’s representatives, *A. baumannii*, is infamous for its involvement in nosocomial infections, such as hospital-acquired pneumonia, bloodstream infections, and wound contaminations. They often affect patients with compromised immune systems, as well as elderly persons and young children [28]. The ability of *A. baumannii* to survive on various surfaces for prolonged periods and its rapid acquisition of mechanisms for antibiotic resistance have provided considerable challenges for pathogenic control and effective treatment [10].

*Acinetobacter haemolyticus* is another reported pathogen known for its ability to cause hemolysis. It has been suspected to cause bloody diarrhea in cases where no other enteropathogenic bacteria were detected [29]. The same species has been isolated from an 86-year-old patient diagnosed with recurrent bronchiectasis.

Other opportunistic pathogens such as *A. lwoffi*, *A. johnsonii* and *A. junii* have been associated with secondary meningitis and bacteremia, among others, although not as frequently as *A. baumannii* [30, 31]. The highly adaptive nature of the genus indicates the need for more data from investigating the potential pathogenicity of non-baumannii species.

On the contrary, research into the environmental and beneficial aspects of *Acinetobacter* spp. has revealed their role in biodegradation and bioremediation processes and their presence in fermented foods. These bacteria are capable of producing a range of enzymes, such as lipases, proteases, and esterases, which are crucial in breaking down complex food substrates into more pure, flavorful compounds [32]. For instance, two *Acinetobacter* strains (*Acinetobacter* sp. 1H8 and *Acinetobacter indicus* 3B2) were inoculated in cigar tobacco leaves, where they not only increased the degradation of macromolecules, produced aldehydes and ketones and increased the content of more flavorful agents, but also promoted the growth of other functional bacteria, such as some *Bacillus* species [33]. *Acinetobacter* also made up the fourth most abundant genera overall in differently sourced da-jiang samples, a traditional soybean fermented food from China, suggesting a high possibility of their active involvement in the product’s processing [34].

**Fig. 2:**
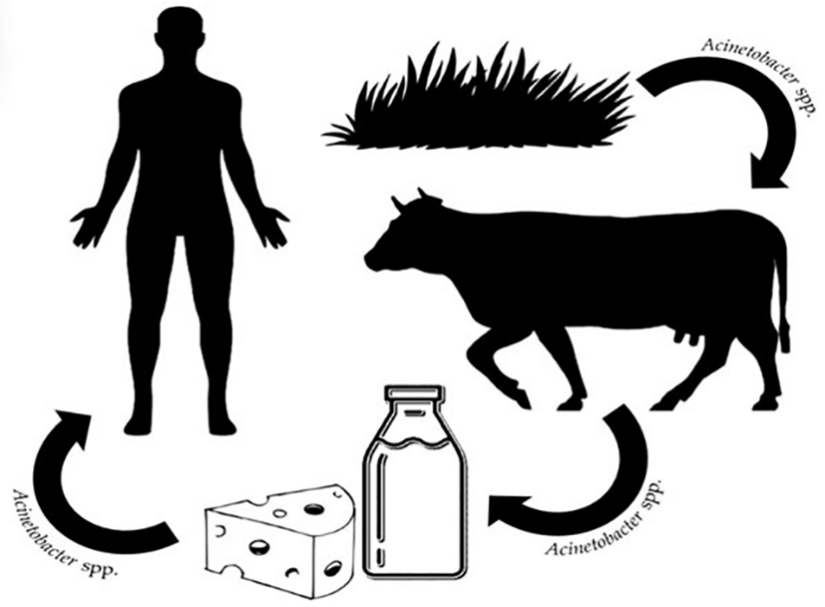
The possible pathway of spreading multidrug-resistant *Acinetobacter* to humans through consuming contaminated food.

However, researchers argue that consuming food in which *Acinetobacter* spp. are present may lead to colonization of the bacteria in the digestive tract and further the spread of multidrug-resistant species (Fig. 2) [35].

## Virulence and Pathogenicity Genetic Determinants

### Antibiotic Resistance Genes

What makes a pathogen a pathogen is a crucial topic for many investigations, primarily those dealing with the nature of microorganisms. A plethora of genetic determinants mainly drives the pathogenicity of *Acinetobacter*, each contributing to the genus’ unique ability to adapt to stress and cause diseases. The most well-known factor is the presence of various antibiotic-resistance genes in their genome, possibly acquired by horizontal transfer of plasmids and transposons from other already resistant microorganisms [36]. For example, the mechanisms of antimicrobial resistance in *A. baumannii* can be grouped into three main categories - the production of antimicrobial-inactivating enzymes, reduced access to bacterial targets, which arises from decreased outer membrane permeability due to loss or diminished expression of porins, as well as the overexpression of multidrug efflux pumps, and mutations that modify targets or cellular functions, such as alterations in penicillin-binding proteins. Non-pathogenic *Acinetobacter* isolates are significantly more susceptible to antibiotics than their pathogenic counterparts [37]. Therefore, the genome of opportunistic pathogens such as *A. baumannii* has a higher content of mobile genetic elements, which promotes the acquisition and the further spread of novel antibiotic-resistance genes [38].

### Error-Prone DNA Polymerases

Additionally, investigations have shown the existence of many error-prone DNA polymerases (EPPs). Within the *Acinetobacter* genus, they may result in point mutations that aid survival, thus furthering their resistance to antibiotics and toxins [21], especially the UmuD’_2_C polymerase (also known in *E. coli* as pol V) [39], associated with a process known as SOS mutagenesis (a process occurring when a cell's SOS response to DNA damage increases the mutation rate) [40]. Interestingly, the SOS response in *Acinetobacter* does not rely on LexA protein, a part of a two-component system that controls the SOS response, which has been proven to be absent within the genus [41]. Most probably, because the expression of the UmuD’_2_C polymerase depends on the unique of the genus non-homologous to LexA proteins UmuDAb and DdR [41, 42], the SOS mutagenesis process is more robust, resulting in gaining resistance to antimicrobial agents under DNA damaging stress caused. This stress can be caused within the clinical environment by antibiotics, UV light and desiccation – practices used in patients’ treatment and disinfection [43].

### Biofilm Formation

*Acinetobacter* representatives such as *A. baumannii*, *A. nosocomialis* and A with *pittii* possess a couple of strategies for successful biofilm formation, which ensures bacterial survival due to the encasing of the colonies with a protective extracellular matrix [44]. They can form on various surfaces in clinical settings and the human body, thus providing a protective environment against antibiotics, cleaning agents and the host immune system. The *csuA/BABCDE* operon encodes components of a chaperone-usher pili assembly system, which is essential for initial attachment and biofilm development. The biofilm-associated protein (Bap) is crucial for biofilm formation on abiotic surfaces. The control of biofilm formation and virulence gene expression is regulated by quorum sensing (QS) systems in response to population density [45]. The ability of *Acinetobacter*, particularly *A. baumannii*, to form such biofilms is one of the leading causes of their infamous pathogenic nature.

### Outer Membrane Proteins

The existence of outer membrane proteins plays a crucial role in bacterial adherence, invasion and immune evasion. The outer membrane protein A (OmpA), a significant meditator of biofilm formation, is one of the most investigated virulence factors of *A. baumannii*, as its overproduction is closely associated with the mortality rate of the nosocomial infections caused by the bacterium [46]. Notably, the initial contact between the pathogen and the epithelial host cell is the binding of OmpA to fibronectin [47]. The protein also modulates the secretion of outer membrane vesicles such as Omp33-36, which also play an essential role in the pathogenicity of *A. baumannii* [48]. The porin is the result of the expression of the *mapA* gene. It instigates apoptosis in infected immune and connective cells by activating caspases and blocking autophagy, thus allowing the bacteria to persist inside autophagosomes [49]. Other distinguished OMPs possessed by pathogenic *Acinetobacter* isolates are CarO, carbapenem susceptibility porin, and OprD, an orthologous protein that plays a role in drug resistance [50].

### Toxins Secretion

Protein secretion systems are detrimental to the possible interactions of bacteria with the environment and host cells. Type II secretion (T2SS) is common in Gram-negative pathogens as it secretes multiple effector proteins such as lipases and proteases, some requiring membrane-bound chaperons to be correctly produced. It has been found that the presence of human serum slightly increased the expression of T2SS in *A. baumannii*, which suggests that environmental factors may positively or negatively influence the virulence of a strain [51]. A clear correlation between the T2SS function and the colonization and infection of mice with *Acinetobacter* has been determined. However, the specific virulence-promoting mechanisms of the secretion system have yet to be pointed out [52]. Type VI secretion (T6SS) is a complex mechanism for injecting toxins directly into contact with competing bacteria, ensuring a possible pathogenic dominance. The genes encoding T6SS in *Acinetobacter* are conservative and are found on a single chromosomal locus. Notably, a non-clinical strain of *A. baumannii*, DSM30011, with a fully active T6SS could eliminate *E. coli*, *Klebsiella pneumoniae*, *Pseudomonas aeruginosa*, and a clinical isolate of *A. baumannii* [53]. Those results hint that while useful in bacterial competition, fully functional T6SS might not be absolutely necessary for pathogenic clinical isolates.

### Siderophores

Another distinguished virulence determinant is the presence of iron acquisition systems. It is well known that iron is essential for bacterial growth and metabolism [54]. *A. baumannii* produces acinetobactin and baumannoferrin, which are siderophores used to sequester iron from host cells because of their high affinity. Their production is mediated by a ferric uptake regulator (Fur), a protein that controls the expression of iron acquisition genes in a metabolic response [55]. Such strategies are crucial for the survival of *Acinetobacter* pathogens inside the animal and human bodies, as they are iron-limited environments [56].

*Acinetobacter* species possess a wide range of virulence factors that enable them to adhere to host cells, form biofilms, and evade immune responses. They provide insights into the complex mechanisms that cause their pathogenicity, which could aid potential disease control strategies. The variety of determinants and genetic differences differentiates a pathogen from a non-pathogen *Acinetobacter* species. Below is a comparison of the genomes between the pathogen *A. baumannii* and the environmental *Acinetobacter baylyi* (Table 1).

## Acinetobacter in the Environment

*Acinetobacter* species possess the capacity to spread on numerous diverse ecological niches, as they have been assessed as microbial weeds [57]. An attempt to explore the distribution of *Acinetobacter* in nature determined that the genus was present in 28 of the 30 unique soil samples and 29 of the 30 unique water samples taken from a vast area along the coast of central California [58]. Aside from being present in differently sourced soil, water, foods and animals, they are also a part of the basic commensal microbiota of healthy humans [59]. The genus’s omnipresence, coupled with its representatives' high adaptability, proposes studying these bacteria as model organisms for environmental, biotechnological and industrial microbiological investigations. For example, *A. baylyi* ADP1, a soil-inhabiting strain, has been found to have the highest research opportunity due to its high transformation and recombination competency, genome plasticity, versatile metabolic abilities, and fast and easy cultivation [60].

### Soil

Members of the genus inhabit a plethora of soil environments. Studies generally focus on their presence in agricultural fields due to both their role in nutrient cycling, specifically in nitrogen fixation and degradation of complex organic elements, and their ability to carry antibiotic resistance genes and virulence-related traits, which in turn could harm the consumers of the eventually produced crops [61]. *Acinetobacter* has been found to be among the dominant genera in the rhizosphere soil of wheat and maize in Turkey. Interestingly, the same investigation concluded that inoculating wheat (*Triticum aestivum*) with *Acinetobacter* sp. WR922, one of the isolated strains, increased the phosphorus content of the plant by 27% on 15th day after the emergence and the dry matter by 15% on the 30th day. These results are attributable to the strain’s phosphorus-solubilizing ability without the need for pyrroloquinoline quinone-like many other phosphate-solubilizing bacteria [62]. Strains of *A. guillouiae* and *A. calcoaceticus* were used as bioinoculants in combination with chemical fertilizers to induce the growth of onion (*Allium cepa*), which in turn grew in length biomass and had a higher availability of valuable compounds, demonstrating *Acinetobacter* representatives as plant growth promoting microbes [63]. Thus, it is suggested that these bacteria contribute to the growth of plants by providing nutrient compounds. Recent findings also demonstrate that *Acinetobacter* could play a role in soil bioremediation through hydrocarbon and phenol biodegradation [27, 64]. For example, petroleum decomposing activities are achieved through the degradation of the C5–C16 alkanes and cycloalkanes by the bacterial P450 oxygenase system, while the C10–C30 alkanes are substrates of the *Acinetobacter*’s dioxygenases [65]. Some examples of isolates with powerful hydrocarbon degrading activity are *A. junii*, which were found to degrade a variety of crude oils, diesel oil, engine oil, fluorene, phenanthrene, docosane and triacontane [66]. Another example is the degradation of the aromatic hydrocarbons by the *Acinetobacter* XS-4, which was proven to be a result of the expression of the salicylate hydroxylase, proacid 3,4-dioxygenase (β subunit) and proacetate 3,4-dioxygenase (α subunit) genes [67].

### Waters

*Acinetobacter* strains are present in both fresh and marine waters as free-living organisms or as biofilms on various surfaces. Such biofilms have been found in drinking water distribution systems, as they are much less susceptible to antiseptics than planktonic *Acinetobacter* species. The genus is found to be the prevalent isolate from chlorinated distributional systems, making up more than 5% of all identified microorganisms [59]. This could result from high resistance to chlorine, the most commonly used water disinfectant, with *A. baumannii* being able to persist in 0.2 to 4 ppm of free chlorine exposure, thus raising concerns about causing potential water-borne diseases [68]. *Acinetobacter* spp. has been identified in 38% of untreated groundwater supplies and 16% of the water supplies, with no total coliforms detected in Northern Preston County. Researchers found no notable difference in slime production, a virulence factor for *A. calcoaceticus*, in drinking water strains and clinical isolates while also highlighting that *Acinetobacter* might mask the presence of total coliforms in water, as some strains are able to interfere with sheen production by a number of coliform bacteria on differential media, which poses a significant threat to the safety of the consumers [69]. Furthermore, the drug-resistant *A. baumannii* ST219 caused an outbreak in Tokai University Hospital's emergency intensive care unit due to the strain's colonization of the water systems, therefore spreading the infection through tap water [70]. Another concern for the genera’s presence in tap water is the possible dissemination of antibiotic resistance to other species [71].

On the contrary, some strains could be a promising alternative to cleaning oil spills and reducing the toxicity of pollutants in wastewater [7]. *Acinetobacter* sp. SCYY-5 has been proven to reduce total petroleum hydrocarbon contamination by 69.17% in 10 days and under optimal degradation conditions by 79.94% in the same period [72]. Another study proposes that *A. junii* strain b2w is highly suitable for chromium bioremediation in contaminated waters since the bacterium could accumulate the heavy toxin effectively without disrupting cell integrity [73]. The positive environmental impact of several *Acinetobacter* strains in waters could be an excellent tool for industrial and biotechnological advancements and thus should be explored further.

### *Acinetobacter* as an Animal Skin Commensal

*Acinetobacter* is considered to be a part of the typical commensal skin microbiota of humans and animals. They have been isolated from multiple animals, including dogs, cats, horses, pigs, and birds [74]. Investigations mainly relevant to veterinary medicine argue that *Acinetobacter* might become an opportunistic animal pathogen and should be given more attention due to the genus’s high affinity for antimicrobial resistance [75, 76]. For instance, a carbapenem-resistant *A. pittii* isolate was detected in a cat skin sample [77]. *A. baumannii* caused necrotizing fasciitis, an infection of the deep layers of the skin and fascia, with septic shock in a domestic shorthair cat, which ended in cardiac arrest [78]. The same has been detected in various animal infection sites, such as chronic eczema and open wounds in dogs [74]. While the presence of pathogenic isolates on the skin of domestic animals is alarming, their detection in livestock appears to be a broader question. A study examined the possible existence of drug-resistant *A. baumannii* in 422 cattle, containing 280 dairy cows, 59 beef cattle, and 83 calves over 14 months. A total of 15.6% of the diverse samples were positive, with dairy cows being the prevalent group with 21.1%, followed by beef cattle (6.8%) and calves (2.4%) [77]. Different farms in Lebanon have been similarly investigated through fecal samples taken from cattle, pigs and hens. Four isolates were inhibited with *A. baumannii*, resistant to a wide range of antibiotics [79]. Tigecycline-resistant A. towneri has been identified in 684 fecal and environmental isolates from six livestock farms, as *Acinetobacter* species appear to be the leading carrier of tigecycline-resistant *tet(X)* genes. Notably, most *tet(X)*-positive isolates seem to be associated with livestock [80]. Such results pose a multitude of speculations about possible contamination of animal foods with virulent *Acinetobacter*, considering that the genus has already been identified in a plethora of such products [2, 35]. Research into the presence of non-pathogenic isolates in animals is severely limited, due to the higher urgency of virulent ones.

## *Acinetobacter* in Fermented Foods

**Fig. 3:**
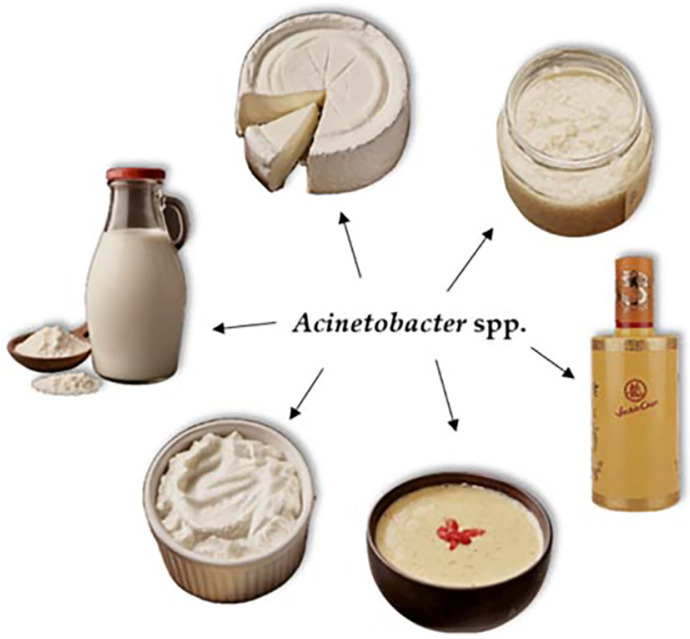
A visual representation of the presence of *Acinetobacter* in fermented foods, all varying in type and origin.

From balancing the gut microbiota and reducing blood pressure to vitamin increase and reduction of inflammation, the numerous health benefits of fermented foods and their content have been extensively studied and promoted; hence, fermented products have gained significant popularity over the years. These qualities are attributed to the bioactive compounds, including vitamins, peptides and polysaccharides, produced by the microbial communities responsible for the fermentation process [81]. Certain *Acinetobacter* representatives have been detected by metagenomic analyses of various fermented foods (Fig. 3). The reasons behind the latter, however, could not be only a result of contamination of the processing environment or the raw material, as consistently mentioned, but also due to the metabolic capacities of the genus that play a practical role during food fermentation. Examples of such capacities are protease and lipase activities, as well as the biosynthesis of small aromatic compounds. Nonetheless, the strains found in food and clinical environments have substantial differences in their genotypes and, therefore, variable metabolic properties [82]. This could possibly impact the finished product in a different way.

### Non-Dairy Fermented Foods

A study exploring the dynamic microbiota of surimi, a protein extract from fish meat during fermentation, concluded that *Acinetobacter* was the second most abundant genera, making up 19.75% and 7.34% of the total bacterial diversity after 36 h and 48 h, respectively. Interestingly, at 0 h, its abundance accounted for 2.02%, then at 12 h – for only 0.99%, before it grew to 12.44% at 24 h. Although the microbiome of the raw ingredient, marine fish, appears to be inhabited mainly by Gram-negative microorganisms, including *Acinetobacter*, the growth of the bacteria in the process could suggest a possible practical activity, even though the problem is left unspecified [83]. *Acinetobacter* has been revealed to be a dominant genus in Daqu, a cereal starter culture needed to produce Baijiu, a Chinese distilled spirit, as it produces esterases, pectinases and lipases, among others, and oxidizes glucose to produce acetic acid. All those components are essential for making the flavorful Baijiu [32]. Similarly, the predominance of the genus was observed in another Chinese product, red sufu, traditional soybean food, where it has been suggested that the bacteria could have a microbiota-stabilizing property [84]. *A. baumannii* TU04 has been isolated from Tapai Ubi, a Malaysian traditional cassava-fermented food, from which researchers identified an *SPSFQ* gene, a determinant of the production of extracellular serine protease that can degrade a variety of tissue-associated protein substrates [85]. *A. lwoffii* has also been identified as the dominant species (4.60%) in fermented rice Bhaati Jaanr, a product with proven antitumor activity [86]. Other fermented foods in which the species have been noticed are chicha, a rice-based fermented beverage; pulque, an alcoholic drink from the Agave plant; chikwangue, a starchy cassava product; koko, fermented maize porridge; and kenkey, steamed dumplings made from a steeping maze in water [87]. The possible functionality of *Acinetobacter* in those foods is scarcely explored, as many attribute its presence to an unsanitary environment.

### Fermented Dairy Foods

*Acinetobacter* strains had a significant abundance in the maturation of two Camembert cheeses, with more than half of the strains presenting lipolytic activities on butterfat and tributyrin agar. Notably, NaCl enhanced the lipase production. No proteolytic strains were detected. In one sample, *A. calcoaceticus* was the most abundant species, apart from lactic acid bacteria. Furthermore, significant growth of *Acinetobacter* was only noticed after the growth of the yeast *Debaryomyces hansenii* [19]. Another study proposed that the genus could possess a secondary activity in the maturation of Istrian cheese [88]. *A. baumannii* has been identified as one species with relative abundance >1% in 92 different spontaneously fermented dairy products, such as shubat, yogurt, butter, sour cream and cottage cheese, all from different Northeast Asian regions [89]. The species was present in 3.3% of overall samples of Domiati cheese, which is attributed to possible inefficient heat treatment or improper handling. In Kareish cheese, another Egyptian product, *A. baumannii*, was detected in 10% of the samples, along with a 3.3% presence of *A. calcoaceticus*, possibly due to the raw milk production process. The latter study also identified *A. baumannii* and *A. haemolyticus* in 13.3% of all cream samples. All of the strains from Domiati and Kareish cheese and three-fourths of those in cream presented positive lipolytic activity, which is argued to potentially reduce the shelf life of the milk products due to a possible production of ropy milk, which is a particular characteristic of the bacteria, although rarely encountered [90, 91]. *A. baumannii* and *A. pittii* have been isolated from raw cheese samples in Lebanon [92]. Furthermore, *A. calcoaceticus*, *A. guillouiae*, *A. johnsonii* and unidentified *Acinetobacter* were all present during the ripening of May bryndza cheese but were absent in the finished product [93]. Similarly to the non-dairy foods, knowledge of the activity of the genus in dairy fermented foods is limited, besides their aforementioned lipolytic and proteolytic ability, which appears to be a characteristic, not all strains possess [3, 91]. It appears that the strains that are present in various products are highly specific, therefore inducing a lack of perceivable trends and broader investigations.

## Conclusion

*Acinetobacter* is a fascinating, highly heterogeneous genus that includes pathogens, biodegradators, stabilizers, model organisms and flavor enhancers. While their virulence is being thoroughly explored, broader and more specific investigations are needed to determine the reason behind their significant presence in a plethora of fermented food products. One way to fill the existing information gap on the genetic bases for biotechnological applications is whole-genome sequencing of non-pathogenic, environmental and fermented foods *Acinetobacter* isolates.

## Figures and Tables

**Table 1:** A comparison of the genomes of *Acinetobacter baumannii* and *Acinetobacter baylyi* in different categories.

	*A. baumannii*	*A. baylyi*	References
Size of genome	Approximately: 3.4 to 4.2 Mb	Approximately: 3.5 Mb	[94, 95]
Mobile genetic elements	Plasmids, transposons, Insertion sequences	More stable genome	[80, 94]
Antibiotic resistance	A Plethora of genes such as *blaOXA-51* and *pmrA*, efflux pumps such as AdeABC, aminoglycoside-modifying enzymes	Lack of many determinants, more susceptible to antibiotics	[12, 94, 96]
Virulence factors	Various factors, such as OmpA, CarO, T2SS and T6SS components	Significantly less or none	[50, 94]
Metabolic adaptability	Equipment for survival in hostile environments	More adaptive and versatile	[94, 97]
Biofilm formation	Bap, *csu* operon, quorum sensing system	Less developed strategies	[44, 98]
Iron acquisition system	Ferric uptake regulator, siderophores - acinetobactin and baumannoferrin	Lack of advanced system	[56, 94]
